# A country-specific FRAX model for Botswana

**DOI:** 10.1007/s11657-021-00965-y

**Published:** 2021-06-07

**Authors:** M. Kebaetse, S. Nkhwa, M. Mogodi, J. Masunge, Y. P. Gureja, M. Ramabu, T. Mmopelwa, I. Sharif, A. Orford, N. C. Harvey, E. V. McCloskey, J. A. Cauley, J. A. Kanis, H. Johansson

**Affiliations:** 1 Faculty of Medicine, University of Botswana, Gaborone, Botswana; 2 Princess Marina Hospital, Gaborone, Botswana; 3 Gaborone Private Hospital, Gaborone, Botswana; 4 Bokamoso Private Hospital, Gaborone, Botswana; 5 MRC Lifecourse Epidemiology Unit, University of Southampton, Southampton, UK; 6 Centre for Metabolic Bone Diseases, University of Sheffield, Sheffield, UK; 7 Mellanby Centre for Musculoskeletal Research, Department of Oncology and Metabolism, University of Sheffield, Sheffield, UK; 8 Department of Epidemiology, Graduate School of Public Health, University of Pittsburgh, Pittsburgh, PA USA; 9 Mary McKillop Institute for Health Research, Australian Catholic University, Melbourne, Australia

**Keywords:** Botswana, Epidemiology, FRAX, Fracture probability, Hip fracture, Major osteoporotic fracture

## Abstract

**Introduction:**

Hip fracture rates in Botswana were used to create a FRAX® model for fracture risk assessment.

**Objective:**

This paper describes the development and characteristics of a country-specific FRAX model for Botswana.

**Methods:**

Age-specific and sex-specific incidence of hip fracture and national mortality rates was incorporated into a FRAX model for Botswana. Ten-year fracture probabilities were compared with those from African countries having a FRAX model and African Americans from the USA.

**Results:**

The probabilities of hip fracture and major osteoporotic fracture were low compared with those from South Africa (Black and Coloured) and US Blacks. Probabilities were marginally higher than for Tunisia.

**Conclusion:**

The creation of a FRAX model is expected to help guide decisions about the prevention and treatment of fragility fractures in Botswana.

## Introduction

In 2008, the then WHO Collaborating Centre for Metabolic Bone Diseases at the University of Sheffield, UK, launched the FRAX® tool for the calculation of 10-year fracture probabilities in women and men from readily obtained clinical risk factors (CRFs) and bone mineral density (BMD) measurements at the femoral neck (http://www.shef.ac.uk/FRAX). The algorithms within (FRAX) were based on a series of meta-analyses of candidate clinical risk factors for fracture using primary data from population-based cohorts that examined a list of candidate clinical risk factors for fracture [1, 2]. The output of FRAX comprises the 10-year probability of major osteoporotic fracture (hip, spine, distal forearm or proximal humerus) or hip fracture alone. This probability is in turn dependent upon the risk of fracture and the competing risk of death, both of which vary from country to country [3]. The availability of FRAX has stimulated studies of fracture incidence that can be used for the generation of new FRAX models; recent examples include Belarus, Kazakhstan, Uzbekistan [4] and, most recently, Botswana [5].

At present, there is a paucity of information about osteoporosis and fractures in Africa [5–13]. FRAX models are only available for Tunisia, Morocco and South Africa. In addition, a surrogate model is available for Zimbabwe based on the South African incidence of hip fracture in the Black community. Recently, the incidence of hip fracture has been reported for the Republic of Botswana [5]. This paper describes the synthesis of a country-specific FRAX model for Botswana.

## Methods

National hip fracture rates were used to populate a FRAX model for Botswana [5]. In brief, in a retrospective population-based survey, hip fractures were identified in 2009, 2010 and 2011 from hospital registers. The age-specific and sex-specific hip fracture incidence rates were then derived and used to construct the FRAX model. For other major osteoporotic fractures (clinical spine, forearm and humeral fractures), it was assumed that the age-specific and sex-specific ratios of these fractures to hip fracture risk found in Sweden were comparable to those in Botswana. This assumption has been used for many of the FRAX models with incomplete epidemiological information. National mortality rates used data from the World Health Organization for 2015–2019 [14].

The development and validation of FRAX have been extensively described [1, 15]. The risk factors used were based on a systematic set of meta-analyses of population-based cohorts worldwide and validated in independent cohorts with over 1 million patient-years of follow-up. The construct of the FRAX model for Botswana retained the beta coefficients of the risk factors in the original FRAX model with the incidence rates of hip fracture and mortality rates for Botswana. Ten-year fracture probabilities were compared to those for countries where a FRAX model was available in Africa (South Africa, Morocco and Tunisia) as well as for US Blacks using the ethnic-specific US FRAX model. For South Africa, we used probabilities for Black and Coloured South Africans. The term ‘coloured’ can be offensive in some parts of the world including the UK (https://www.bbc.co.uk/news/newsbeat-54888197). In South Africa, however, the term ‘coloured’ is used to denote multiple heritages and is used in this context.

Additionally, summary 10-year probabilities were standardised to the world population from the age of 50 years for 2020 using the medium variant for fertility [16].

## Results

The 10-year probabilities of major osteoporotic fracture and hip fracture in Botswana and other African countries are shown in Table 1 in women with a prior fracture by age. For both hip fracture and major osteoporotic fracture, 10-year probabilities were the lowest for Botswana and Tunisia, intermediate for Morocco and South African Blacks, and the highest for South African Coloureds and US Blacks. The increase in fracture probability with age was less marked in Botswana and other African countries than in US Blacks. For example, the probability of hip fracture at 80 years was 7.5-fold higher than that at 50 years in Botswana, whilst the comparable ratio was 17.7 in US Blacks. There was a greater than threefold difference in fracture probabilities between the highest and the lowest probabilities at any given age across the countries.

**Table 1:** A 10-year probability of a major osteoporotic fracture (MOF) and hip fracture in women with a prior fracture by age from Botswana, South Africa, Morocco and US Blacks. Body mass index is set to 25 kg/m^2^

Age (years)	Botswana	South Africa, Black	South Africa, Coloured	Morocco	Tunisia	US Black
MOF						
50	1.6	2.6	3.1	2.1	1.0	3.5
55	1.7	3.1	4.6	3.1	1.2	5.3
60	1.7	3.7	6.3	4.5	1.6	6.9
65	1.7	4.3	7.6	5.8	2.0	8.2
70	1.9	5.1	9.1	7.0	2.3	9.9
75	2.3	6.2	11	8.2	2.4	12
80	2.7	7.2	13	9.1	2.6	16
85	3.3	8.7	16	10	3.0	18
90	3.8	9.8	18	10	3.4	16
Hip						
50	0.2	0.5	0.5	0.3	0.1	0.3
55	0.2	0.7	0.9	0.6	0.2	0.5
60	0.3	1.0	1.4	1.0	0.4	0.9
65	0.5	1.4	2.1	1.6	0.6	1.4
70	0.8	1.9	2.9	2.2	0.7	2.2
75	1.1	2.3	3.7	2.9	0.8	3.6
80	1.5	2.7	4.7	3.7	1.0	5.3
85	1.7	3.6	6.9	5.1	1.2	6.6
90	1.5	4.4	9.3	6.4	1.4	6.3

**Fig. 1:**
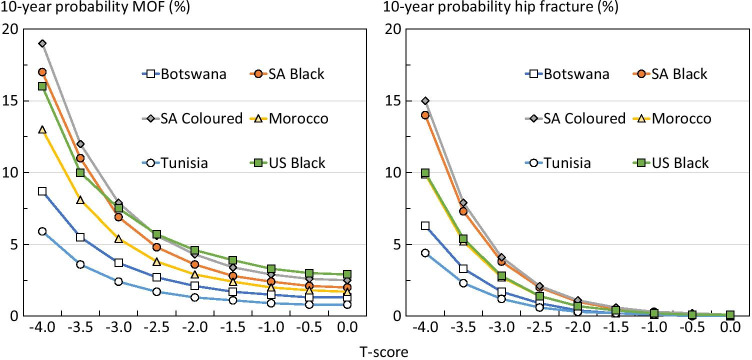
A 10-year probability of a major osteoporotic fracture and hip fracture in women age 50 years from diverse populations by T-score for femoral neck BMD. Body mass index is set to 25 kg/m^2^ with no other clinical risk factors (SA, South Africa; US, United States)

A similar rank order of probability was seen over a wide range of BMD shown for women age 50 years in Fig. 1.

**Fig. 2:**
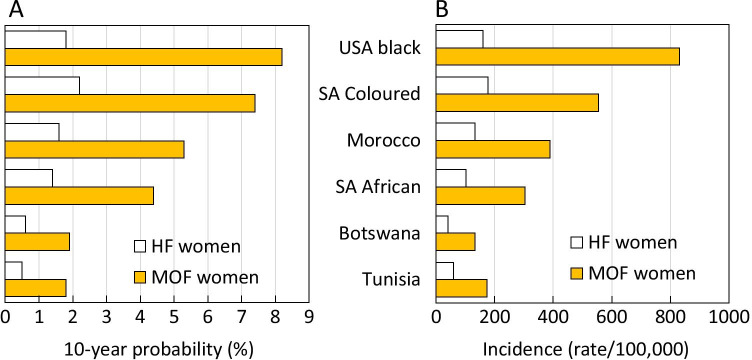
Age-adjusted 10-year probability of hip fracture (HF) and major osteoporotic fracture (MOF) in the female populations studied (**A**) and age-adjusted incidence in the same populations (**B**). Adjustments were to the world population 2020

The rank order of 10-year probability after age adjustment is shown for women in Fig. 2 when standardised to the world population from the age of 50 years for 2020. For major osteoporotic fracture, the highest probability was seen for the US Blacks followed by Coloureds from South Africa. Intermediate rates were seen for Morocco and South African Blacks and the lowest rates for Botswana followed by Tunisia. The rank order reflected in large part the rank order of fracture incidence. Similar findings were noted in men.

## Discussion

The incidence of hip fracture and of death was used to create a country-specific FRAX model for Botswana. Ten-year probabilities of fracture were consistently lower than in the neighbouring country of South Africa, lower than probabilities for Morocco and US Blacks but slightly higher than those for Tunisia. These differences in fracture probability largely reflected differences in the age-specific risk of hip fracture (see Fig. 2). Reasons for the heterogeneity in hip fracture risk are speculative [17] and include hip geometry, ethnic-specific factors, exposure to risk factors such as sunlight (geographical latitude), vitamin D deficiency, duration of fertile life, risk of falling and prenatal nutrition, all of which could affect the risk of fractures in later life. Lifestyle factors such as physical activity, diet and smoking might also contribute [18–20]. Ecological studies do not suggest important roles for these risk factors with the possible exception of physical activity on the risk of falling [21]. The factor which best predicts the heterogeneity in hip fracture risk is socioeconomic prosperity that in turn may be related to low levels of physical activity [22]. Conversely, the high levels of physical activity, especially in rural areas, may contribute to the low fracture probabilities in Botswana. The fact that there are differences in adjacent countries emphasises the importance of the use of country-specific FRAX models where available rather than surrogate models [23]. Although fracture rates have been measured in the Black population of the USA [23], there is little information to determine whether these are comparable to rates in Africa. The present study suggests that fracture rates are lower in Blacks from Africa than that in the USA, again for reasons that are speculative.

There is an interesting disparity between fracture incidence and fracture probability in Botswana and Tunisia. Hip fracture rates were lower in Botswana than in Tunisia, but fracture probabilities were higher in Botswana than in Tunisia. The contrast is due to the lower mortality in Tunisia than Botswana. These observations emphasise the importance of the death hazard as well as the fracture hazard in the determination of fracture probability.

A minority of countries that have a FRAX model also have robust information on the risk of other major osteoporotic fractures. In the absence of such information, FRAX models are based on the assumption that the age-specific and sex-specific pattern of these fractures is similar to that observed in Malmo, Sweden [24]. The assumption has been validated in studies from Canada [25], Iceland [26], the USA [27], the UK [28], Australia [29] and Eurasia [30] despite differences in incidence [3, 18]. This commonality of pattern is supported by register studies, which indicate that in those regions where hip fracture rates are high, so too is the risk of forearm fracture and spine fractures (requiring hospital admission) [31, 32]. Studies of incidence rather than prevalence of vertebral fracture confirm a much higher incidence of vertebral fracture in US Blacks than Whites [33].

**Fig. 3:**
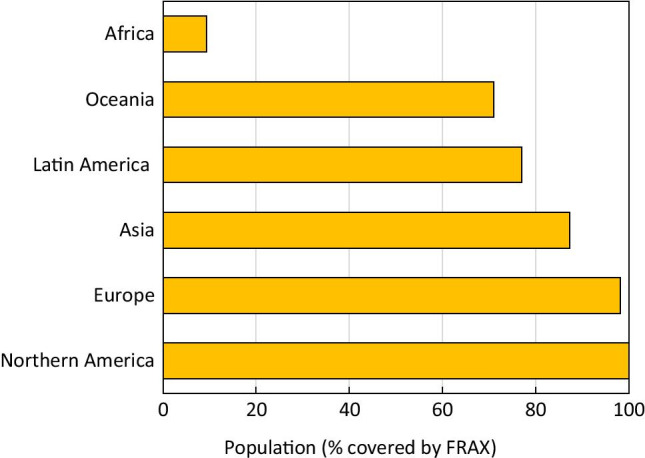
The proportion of the total population served by FRAX in each WHO region of the world

A notable gap in the availability of FRAX models is evident in Africa. FRAX models are available for South Africa, Morocco and Tunisia and now for Botswana but none for the other 50 African Nations. A surrogate model has recently been released for Zimbabwe. However, only 9.3% of the population in Africa have a country-specific FRAX model. This compares with a high coverage in Europe (98%), Northern America (100%), Latin America (77%) and Oceania (71%) (Fig. 3).

In most countries, a case finding approach is used for the management of osteoporosis, where certain clinical risk factors (CRFs) for fracture suggest the possible diagnosis of osteoporosis and trigger a more detailed assessment of the need for intervention. Many assessment guidelines recommend that women with a prior fracture are eligible for treatment. By the same token, individuals with a fracture probability that is equivalent to or greater than that of women of the same age with a prior fracture should also be eligible. Age-specific intervention thresholds have been widely used in Europe and South America [34]. If the same strategy were used in Botswana, intervention would be recommended in individuals with a 10-year probability of a major osteoporotic fracture that ranged from 1.6 to 3.8%, depending on age (see Table 1).

The limitations of the present study relate predominately to the accuracy of each FRAX model studied. This in turn is dependent on the accuracy of the fracture and death hazards used in the construction of the various FRAX models. Whereas death rates (with the exception of South Africa) are from the same source (WHO), fracture rates are variously determined with uncertain precision errors. Notwithstanding, the probability estimates are consistently low in all the populations studied. Age-standardised FRAX probabilities for a major osteoporotic fracture varied approximately fourfold between the populations studied. Much greater heterogeneity in fracture risk is observed worldwide [3].

In conclusion, this paper describes the development of the FRAX model for Botswana which allows the estimation of 10-year probability of hip and major osteoporotic fracture in residents of Botswana. The calibrated model is based on the original FRAX methodology, which has been externally validated in several independent cohorts.
